# Understanding Glaucoma One Synapse at a Time

**DOI:** 10.1523/ENEURO.0118-23.2023

**Published:** 2023-05-08

**Authors:** Emily Winson-Bushby, Ana Dorrego-Rivas

**Affiliations:** Centre for Developmental Neurobiology, Institute of Psychiatry, Psychology and Neuroscience, King’s College London, London SE1 1UL, United Kingdom

Glaucoma is a neurodegenerative condition in which increased intraocular pressure (IOP) causes progressive degeneration of retinal ganglion cells (RGCs), the neurons projecting from the retina to secondary visual areas of the brain. Raised IOP results in apoptosis of RGCs because of damage to RGC axons at the optic nerve head. Axonal damage triggers disruption of RGC mitochondria, metabolism, and active transport. It also disturbs the anatomic distribution of excitatory connections in RGC targets, including the superior colliculus and the thalamic dorsal lateral geniculate nucleus (dLGN). However, the timeline of the processes linking initial damage to RGC death is not well characterized. It is especially unclear at which point functional changes begin in excitatory synapses between RGCs and target structures like the thalamocortical (TC) neurons in the dLGN. To solve this conundrum, Smith et al. (2022) established a timeline for the pathologic processes induced by raised IOP by studying the RGC to TC synapse in the DBA/2J (D2) mouse, a well-known model of inherited glaucoma.

After establishing the age of onset of IOP elevation at approximately seven months in D2 mice, the authors first measured anterograde axonal transport along RGCs to the dLGN using injection of fluorescently tagged cholera toxin (CTb-594) into the eye. D2 mice showed a progressive decline of anterograde transport, which was significant in the nine-month age group. Similarly, D2 mice showed progressive loss of glutamatergic RGC presynaptic terminals in the dLGN, as indicated by a decreased density of immunolabelled vGLUT2 puncta. This effect was significant by nine months of age, was severe by 12 months, and occurred to a greater extent in areas of the dLGN with impaired axonal transport as determined by the CTb-594 assay.

Smith and colleagues then sought to investigate changes in functional synaptic transmission at the RGC-TC synapse using whole-cell electrophysiology in acute slices. Spontaneous EPSCs (sEPSCs) in TCs did not display changes in amplitude but had a decreased frequency at nine months of age. This effect suggests that a proportion of synapses had been lost as opposed to individual synapses being individually weakened. Next, the authors assessed the paired pulse ratio (PPR), which relates inversely to presynaptic vesicle release probability (P_r_), and the AMPA/NMDA ratio, which indicates the relative activations of postsynaptic AMPA receptors (AMPARs) and NMDA receptors (NMDARs). They discovered a progressive increase in the PPR, indicating a falling P_r_, and coincident decreases in both the AMPAR and NMDAR components of EPSCs, but no changes in the overall AMPA/NMDA ratio. These findings point toward a presynaptic locus of effect for the reduction in synaptic transmission, indicating that the release probability in RGCs terminals progressively decreases with age, but finding no evidence that postsynaptic sites on TCs change in receptor composition. Using a reduced stimulus to evoke single-axon synaptic vesicle release from RGCs (single fiber EPSC), the authors found that the EPSC amplitude recorded in TCs was unchanged. However, the ratio of the single fiber EPSC to the maximal EPSC was increased in the 12-month-old group, suggesting that TC neurons in D2 mice receive overall less inputs than the controls. In summary, the effects observed in both spontaneous and evoked synaptic transmission converge onto a pathologic change in transmitter release at the RGC to TC synapse.

TCs are known to be affected in the context of elevated IOP, with defects in intrinsic excitability and soma atrophy (Van Hook et al., 2020). To investigate potential postsynaptic defects in TCs in D2 mice, Smith and colleagues evaluated dendritic morphology in control and 9- and 12-month-old D2 mice. Sholl analysis revealed a subtle loss in dendritic complexity in the 12-months group, with a reduced number of intersections, specifically those that are closer to the soma. Previous studies have reported that this region receives more RGC inputs as compared with distal dendrites (Morgan et al., 2016), and it is known that synaptic inputs are crucial for dendritic maintenance (Lin and Koleske, 2010). Therefore, the authors hypothesize that dendritic complexity loss in D2 mice is a response to the RGC to TC synapse dysfunction rather than one of the causes.

Finally, even if the axonal defects are well advanced in 11- and 12-months D2 mice, the density of RGC somas in the central and peripheral retina was unchanged, showing that at this stage pathology has not yet caused cell loss. RGC degeneration is rather a late event in the D2 mouse model, starting after 15 months of age (Buckingham et al., 2008).

In conclusion, this study uses an elegant combination of anatomic and physiological approaches to describe the progressive dysfunction of the retinogeniculate synapse in the D2 mouse model of glaucoma (Fig. 1). Some of the anatomic findings reported here, such as the loss of axonal anterograde transport, were also described in the superior colliculus in the same mouse model (Crish et al., 2010), providing new insights into common pathologic outcomes involving different parts of the visual system. The electrophysiological work in this study is outstanding, showing strong evidence of a functional loss of RGC output synapses onto TC in glaucoma.

**Figure 1.**
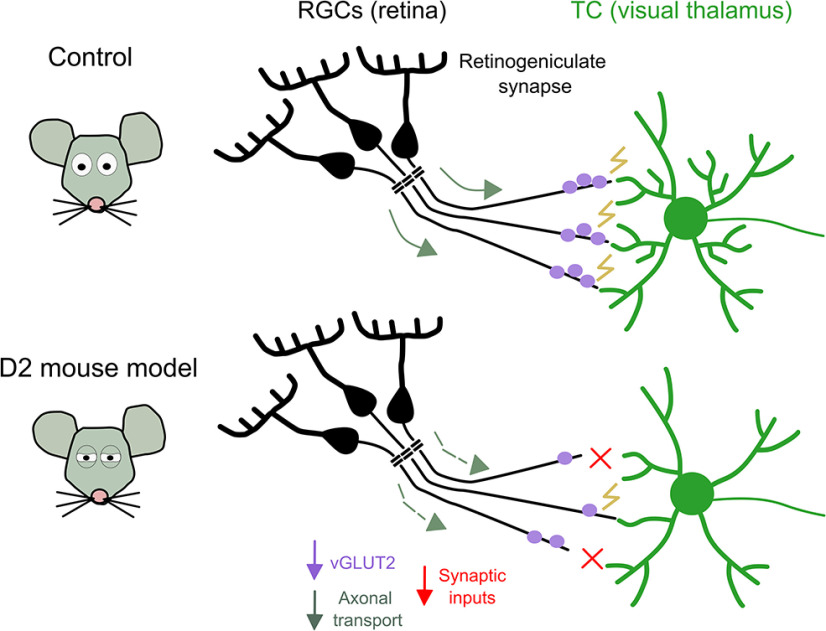
Structural and functional defects of the retinogeniculate synapse in the D2 mouse model of glaucoma. In D2 mice, RGC axons show impaired axonal transport and decreased vGLUT2 staining in the presynaptic terminals. Functionally, the RGC to TC synaptic transmission is reduced because of a lower number of RGC inputs onto TCs. TC neurons have reduced dendritic complexity, with a loss of ramifications specifically in processes that are closer to the soma. RGCs = retinal ganglion cells, TCs = thalamocortical neurons.

As emphasized in the paper’s Discussion, some points remain to be answered considering these results. For example, it is still unclear if decreased vGLUT2 staining is caused by degenerating RGC-to-TC synapses or by defects in axonal transport. In addition, knowing if dendritic remodeling in TCs is followed, or alternatively preceded, by a loss of postsynaptic markers will be key to understanding the pathologic processes operating at retinogeniculate synapses. To conclude, the authors highlight an interesting paradox: while this study shows decreased synaptic transmission between RGCs and TCs, they reported increased TC excitability in D2 mice in previous work (Van Hook et al., 2020). Smith and colleagues suggest an interesting perspective, where the higher excitability of TCs is a homeostatic mechanism to ensure the transmission of visual information until the disease has progressed to a point where this transmission is entirely compromised.

This study adds unprecedented value to glaucoma research. Further work describing cellular and molecular mechanisms behind retinogeniculate synapse dysfunction will be essential to fully understand the pathologic underpinnings of this disease.

## Synthesis

Reviewing Editor: Matthew Grubb, King’s College London
